# The Tenth International Medical Congress

**Published:** 1889-10

**Authors:** 


THE TENTH INTERNATIONAL MEDICAL CONGRESS.—The following notice has been issued:

We, the undersigned, do hereby give notice that, according to the resolution passed at the Washington meeting, September 9, 1887, the Tenth International Medical Congress will be held in Berlin. The Congress will be opened on the fourth and closed on the ninth day of August, 1890. Detailed information as to the order of proceedings will be issued after the meeting of the delegates of the German medical faculties and medical societies at Heidelberg, on the 17th of September in the current year. Meanwhile, we should feel sincerely obliged if you would kindly make this communication known among your medical circles, and add, at the same time, our cordial invitation to the Congress.

von BERGMANN,
VIRCHOW,
WALDEYER.

